# Spectrally and Time-Resolved Fluorescence Imaging of 22-NBD-Cholesterol in Human Peripheral Blood Mononuclear Cells in Chronic Kidney Disease Patients

**DOI:** 10.3390/molecules26226800

**Published:** 2021-11-11

**Authors:** Ingrid Lajdova, Livia Ovsonkova, Viera Spustova, Adrian Oksa, Dusan Chorvat, Anton Mateasik, Alzbeta Marcek Chorvatova

**Affiliations:** 1 Department of Clinical and Experimental Pharmacology, Faculty of Medicine, Slovak Medical University, 83303 Bratislava, Slovakia; ingrid.lajdova@szu.sk (I.L.); viera.spustova@szu.sk (V.S.); adrian.oksa@szu.sk (A.O.); 2 Department of Biophotonics, International Laser Centre of the Slovak Centre of Scientific and Technical Information, 84104 Bratislava, Slovakia; livia.ovsonkova@gmail.com (L.O.); dusan.chorvat@cvtisr.sk (D.C.); anton.mateasik@cvtisr.sk (A.M.); 3 Department of Biophysics, Faculty of Natural Sciences, University of Ss. Cyril and Methodius, 91701 Trnava, Slovakia

**Keywords:** 22-NBD-cholesterol, CKD—chronic kidney disease, FLIM—fluorescence lifetime imaging microscopy, PBMC—peripheral blood mononuclear cells, spectrally resolved confocal microscopy, statins

## Abstract

The interaction of the fluorescent probe 22-NBD-cholesterol with membranes of human peripheral blood mononuclear cells (PBMC) was tested by time- and spectrally resolved fluorescence imaging to monitor the disturbance of lipid metabolism in chronic kidney disease (CKD) and its treatment with statins. Blood samples from healthy volunteers (HV) and CKD patients, either treated or untreated with statins, were compared. Spectral imaging was done using confocal microscopy at 16 spectral channels in response to 458 nm excitation. Time-resolved imaging was achieved by time-correlated single photon counting (TCSPC) following excitation at 475 nm. The fluorescence of 22-NBD-cholesterol was mostly integrated into plasmatic membrane and/or intracellular membrane but was missing from the nuclear region. The presence of two distinct spectral forms of 22-NBD-cholesterol was uncovered, with significant variations between studied groups. In addition, two fluorescence lifetime components were unmasked, changing in CKD patients treated with statins. The gathered results indicate that 22-NBD-cholesterol may serve as a tool to study changes in the lipid metabolism of patients with CKD to monitor the effect of statin treatment.

## 1. Introduction

The NBD (7-nitrobenz-2-oxa-1,3-diazol-4-yl) group [1] is a fluorophore used in biophysical, biochemical and biological studies of cell membranes. NBD-labeled lipids are extensively used as fluorescent analogues of native lipids in biological and model membranes to monitor a variety of processes. As early as in 1996, Mukherjee and Chattopadhyay [2] described a component of the fluorophore 22-NBD-cholesterol in their work on model membranes, which had a maximum fluorescence at 522 nm. The component was evenly distributed throughout the membrane bilayer, and based on its properties, was found to be a monomeric form of the fluorophore. The second component with a maximum at 539 nm was observed after increasing the concentration of the NBD probe, and based on the observed absorption and fluorescence properties, was attributed to the dimerization of the NBD probe. Others later confirmed the presence of the two spectral forms [2,3,4,5,6], as well the segregation of the fluorophore into domains to form dimers [3].

Finding new tools for study of the lipid metabolism and the efficacy of statin therapy in patients with CKD is still highly demanded to complement the existing biochemical analysis. CKD is a worldwide public health problem: recent epidemiological studies from several countries confirmed the prevalence of CKD between 10% and 13% [7]. CKD is a state of gradual loss of kidney function that is often associated with the presence of other civilization diseases such as cardiovascular diseases (CVD), diabetes mellitus, hypertension, dyslipidemia and others [8,9]. Disturbances in lipoprotein metabolism are evident even at the early stages of CKD. Patients with CKD often have low high-density lipoprotein (HDL) cholesterol, normal or low total cholesterol (TC), low-density lipoprotein (LDL) cholesterol and increased triglycerides (TG). [10,11,12,13]. Although LDL cholesterol is not usually elevated in patients with CKD, LDL particles tend to be smaller, denser and more atherogenic in their form. Oxidized LDL cholesterol and intermediate-density lipoprotein (IDL) cholesterol, which are considered to be highly atherogenic, are increased [14]. HDL cholesterol may be dysfunctional, becoming pro-inflammatory and losing its atheroprotective ability to promote cholesterol efflux from cells. Recently published studies indicate that dyslipidemia in CKD patients may actively participate in the pathogenesis of CVD, as well as in the deterioration of renal function [15,16].

PBMC are easily accessible cells from the patients’ blood and thus represent a good target for an additional analytical methodology. Monitoring of 22-NBD-cholesterol’s interaction with PBMC membranes using its optical properties could improve the diagnostics of dyslipidemia in CKD and/or the effectiveness of treatment with various types of statins. In the last decades, the combination of the time-resolved fluorescence techniques with imaging, known as fluorescence lifetime imaging microscopy (FLIM), has been demonstrated as a perspective tool for non-invasive investigations of fluorescence changes in living systems [17]. This is, in part, also due the fact that fluorescence lifetimes are particularly sensitive to the fluorophore environment [18].

In this study, we evaluate the possible employment of the 22-NBD-cholesterol incorporated in human PBMC membranes in the investigation of the presence of CKD with or without statin therapy by means of spectrally and time-resolved fluorescence characteristics of the probe.

## 2. Materials and Methods

### 2.1. Subjects

The subjects of this study comprised 12 patients with CKD stage 3–5, including 5 without statin treatment and 7 treated with statins, and 6 healthy volunteers (HV) with normal hematological and biochemical parameters were included. In this pilot study, patients with varying degree of renal impairment (excluding renal replacement therapy) participated. All subjects were screened and followed up in the outpatient department of nephrology at the Slovak Medical University in Bratislava. The diagnosis of CKD was based on clinical and laboratory examinations as defined by the Kidney Disease Improving Global Outcomes (KDIGO) guidelines [19]. The glomerular filtration rate (GFR) was estimated by the CKD EPI formula [20]. Exclusion criteria for the study were acute impairment of renal function, nephrotic proteinuria, polycystic kidney disease, malignancies, systemic inflammatory conditions and hematologic and autoimmune diseases. Patients receiving corticosteroids, immunosuppressive or immunostimulant drugs were also excluded. The Ethics Committee of the Slovak Medical University approved the study, and all participants gave their written informed consent.

### 2.2. Reagents

The physiological salt solution contained (mmol/L) 140 NaCl, 5.4 KCl, 1 CaCl_2_, 1 Na_2_HPO_4_, 0.5 MgCl_2_, 5 glucose and 5 HEPES (pH = 7.4). The osmolality of physiological salt solution was 290–300 mOsm/kg (Advanced Micro Osmometer 3300, Advanced Instruments, Inc., Norwood, MA, USA). The lymphocyte separation medium Histopaque^®^-1077 was from Sigma-Aldrich (St. Louis, MO, USA), and Digitonin was from Fluka (Buchs, Switzerland). Trypan Blue and 22-(7-nitrobenz-2-oxa-1,3-diazol-4-yl-amino)-23, 24-bisnor-5-cholen-3-ol (22-NBD-cholesterol) were purchased from Thermo Fisher, Invitrogen (Waltham, MA, USA). All other chemicals were obtained from Sigma-Aldrich (St. Louis, MO, USA).

### 2.3. Analytical Procedures

Total cholesterol (TC), HDL cholesterol, TG, CRP and creatinine were measured on the Vitros 250 Analyzer (Johnson & Johnson, Rochester, NY, USA) in serum samples. LDL cholesterol and very low-density lipoprotein (VLDL) cholesterol were calculated according to known formulas [21]. The routine hemogram was measured in whole blood samples using the hematology analyzer Sysmex XS-1000i (Sysmex Corporation, Kobe, Japan).

### 2.4. Isolation of Human Peripheral Blood Mononuclear Cells (PBMC)

Human PBMC were isolated from heparinized blood by Ficoll gradient centrifugation, diluted 1:1 with physiological salt solution, layered on to an equivalent volume of medium Histopaque-1077 and centrifuged at 700 *g* for 20 min at 22 °C, as previously reported [22]. The PBMC layer was removed and washed in 40 mL physiological salt solution, resuspended in 10 mL physiological salt solution and centrifuged at 700× *g* for 10 min at 22 °C. The pellet was resuspended in 2 mL aliquots of physiological salt solution [23]. Our preparations contained lymphocytes (90%–94%), monocytes (3%–6%) and NK cells (the rest), as determined by a flowcytometry (Cytomics FC500, Beckman Coulter, CA, USA). The cell viability was quantified with 0.4% trypan blue solution and estimated to be approximately 98%.

### 2.5. Fluorescence Staining and Incorporation of Membrane Cholesterol

The population of 2 × 10^6^ cells/mL was loaded with 22-NBD-cholesterol at a final concentration of 16 μmol/L (stock solution 8 mmol/L dissolved in DMSO) for 1 h at 22 °C in the dark. After incubation, the cells were centrifugated at 700 *g* for 4 min at 22 °C. The supernatant was removed, and the pellet was dissolved in physiological salt solution [23]. Samples were analyzed by confocal microscopy and FLIM.

### 2.6. Confocal Microscopy

The confocal microscope LSM 510 Meta (Zeiss, Jena, Germany) with a C-Apochromat 40× NA = 1.2 water immersion objective was used for confocal imaging of the samples containing 22-NBD-cholesterol in response to 458 nm excitations. The emission signal was detected across 16 spectral channels covering the spectral region from 472 nm to 643 nm with the step of 11 nm. The spectral emission profile of individual cells was built by summing of spectral profiles from each pixel attributed to the cell body. It typically showed two dominant peaks with varying intensities between different cells and samples. Automatic component extraction (ACE) offered by ZEN 2009 software package (Zeiss, Germany) was used to estimate the two spectral profiles R_1_(λ) and R_2_(λ) present in the measured images. To obtain the shapes of R_1_(λ) and R_2_(λ), respectively, the ACE method was applied to a set of 10 arbitrarily selected spectral images, and the final shapes of R_1_(λ) and R_2_(λ) used later in the analysis were estimated by averaging of respective components found in the individual images. These extracted spectral profiles subsequently served as reference spectra for the data analysis. Each of the estimated spectral profiles showed only one spectral maximum, namely R_1_(λ) at 520 nm and R_2_(λ) at 552 nm. Spectral decomposition [24] was applied to the spectral profile built for each cell segmented from the recorded spectrally resolved image, to obtain the proportion of R_1_(λ) and R_2_(λ). The segmentation of cells was done manually by delineating the area of each cell recorded in the images. The spectral profile was then expressed as a linear combination of reference spectra R_1_(λ) and R_2_(λ), with corresponding proportion weights c_1_ and c_2_, respectively_._

S_theor_ (λ) = c_1_ × R_1_(λ) + c_2_ × R_2_(λ) (1)

All calculations were done using Excel and its optimization module Solver (Microsoft, Redmond, WA, USA).

### 2.7. Fluorescence Lifetime Imaging Microscopy (FLIM)

FLIM by time-correlated single photon counting (TCSPC) was applied to monitor an interaction of 22-NDB-cholesterol with PBMC. FLIM images were recorded by LSM 510 Meta microscope (Zeiss, Germany) euipped with a C-Apochromat 40× N_A_ = 1.2 water immersion objective in response to excitation with a picosecond laser operating at 475 nm nm and LP 505 with 20 MHz pulse repetition. The emitted fluorescence was detected by a photomultiplier HPM 100-40. Obtained FLIM images were segmented by thresholding to delineate each cell present in image. The fluorescence signal from image pixels attributed to the area of each cell was summed and used for the calculation of fluorescence decay characteristics for each cell individually. The fitting procedure assumed the double exponential model, expressed by following equation.

f(t) = amp(1) × exp^(−t/^^τ^^(1))^ + amp(2) × exp^(−t/^^τ^^(2))^ + f_0_ (2)

where τ(i) is lifetime of i-th component with amplitude of amp(i). Fitting was done in the MicroSpace application (ILC CVTISR Bratislava, Slovakia) that exploits the NLOPT numerical library for non-linear regression. From both fluorescence lifetimes and corresponding amplitudes, the mean lifetime τ was determined.

t_m_ = τ(1) × amp(1) + τ(2) × amp(2) (3)

### 2.8. Statistical Analysis

The recorded confocal data were processed using Zeiss LSM Image Browser Version 4.2.0.121 and/or Zeiss ZEN 2009 (Carl Zeiss MicroImaging, Jena, Germany). FLIM data were analyzed by SPCImage Version 5.0 (Becker and Hickl, Berlin, Germany) and MicroSpace (ILC CVTISR Bratislava, Slovakia), which used the NLOPT numerical library (non-linear optimization) for FLIM data analysis [25]. The statistical analysis was carried out by the IBM SPSS version 21 (SPSS Inc., Chicago, IL, USA). Results are presented as means ± SD.

To test the normality of the group distribution, the Shapiro–Wilk test was used. The statistical significance of differences was tested by the independent two-population Student’s *t*-test for normally distributed data and the Wilcoxon’s test for not normally distributed data for a non-parametric analysis between group pairs. A *p*-value < 0.05 was considered statistically significant.

## 3. Results

### 3.1. Clinical and Biochemical Parameters

Selected clinical and biochemical parameters are listed in the Table 1. Healthy volunteers (HV) had all parameters in the reference range. CKD patients without statin treatment had elevated serum LDL cholesterol, VLDL cholesterol and TG concentrations, and decreased serum HDL cholesterol. In CKD patients treated with statins, TC and HDL cholesterol were reduced, while the concentration of LDL cholesterol was at the lower limit of the reference range. VLDL cholesterol and TG concentrations were still elevated in these patients. Patients treated with statins were of more advanced CKD with lower mean eGFR. Mean CRP values were significantly increased (although still within the reference range) in all patients with CKD in comparison with HV. Recorded biochemical parameters confirm the efficiency of statin treatment in the group of patients selected for evaluation of the optical properties of 22-NBD-cholesterol.

### 3.2. Fluorescence of 22-NBD-Cholesterol in PBMC

The fluorescence intensity of the 22-NBD-cholesterol was monitored in PBMC using confocal microscopy. Our preparation contained 90–94% of lymphocytes (other cells included monocytes and NK cells) but, as we are working on individual cells, data gathered in this study were recorded predominantly on this cell type and no other cell types were taken into account.

**Figure 1:**
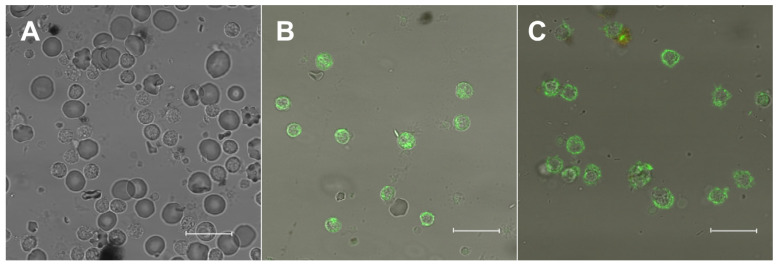
Confocal imaging of cells stained with 22-NBD-cholesterol. Comparison of confocal images of (**A**) unstained cells, (**B**) 22-NBD-cholesterol-stained cells in the absence of digitonin from HV and (**C**) in the presence of digitonin from HV. Excitation laser wavelength 458 nm, C-Apochromat 40×/1.2 W corr lens, filters: Ch2-BP500-550IR, Ch3-BP 650-710IR. Scale: 10 μm.

Unstained cells (Figure 1A) showed no endogenous fluorescence when recorded with the same settings. PBMC exposed to 22-NBD-cholesterol (Figure 1B) presented intracellular and plasma membrane staining that was lacking in the nuclear region. When digitonin, which is capable of perforating cells at the cholesterol sites [26], was added to cells, we observed a clear change in the spatial distribution of the 22-NBD-cholesterol in PBMC (Figure 1C). In its presence, most of the 22-NBD-cholesterol staining was found in the outskirts of the cell, with little or no intracellular distribution, proving the incorporation of the 22-NBD-cholesterol in control conditions.

### 3.3. Spectrally Resolved Analysis of 22-NBD-Cholesterol in PBMC

**Figure 2:**
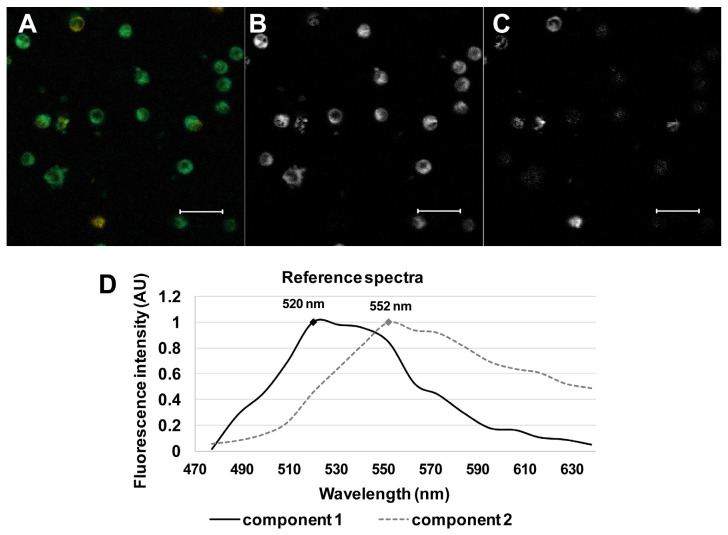
Spectrally resolved imaging of cells stained with 22-NBD-cholesterol. (**A**) Spectrally resolved image of cells recorded at a wavelength of 472–643 nm from HV. (**B**) Distribution of R_1_(λ)with emission maximum 520 nm and (**C**) distribution of R_2_(λ) with emission maximum 552 nm gathered after spectral decomposition. Scale: 20 µm. (**D**) Reference spectra used for the spectral decomposition.

Spectral characteristics of the 22-NBD-cholesterol were evaluated using spectrally resolved confocal microscopy. Spectrally resolved images of the PBMC cells stained with 22-NBD-cholesterol were taken between 472 and 643 nm (Figure 2A). The gathered images showed maximal fluorescence in the spectral region from 530 nm to 550 nm. The linear unmixing approach, described in [24], provided the proportion weights c_1_ and c_2_ of reference spectral profiles R_1_(λ) and R_2_(λ) for each cell, respectively (Figure 2). Two individual spectral components were thus unmasked: the reference spectra with the proportion weight c_1_ had a maximum at 520 nm (Figure 2B,D, black), and that of the c_2_ had a maximum at 552 nm (Figure 2C,D, dashed grey). The presence of the two spectral components is in agreement with previous findings [2,3,4,5,6] and may indicate the presence of monomeric vs. dimeric forms [2]. The more abundant c_1_ component points to the presence of the monomeric form, while the c_2_ may rather correspond to the dimeric one.

### 3.4. Comparison of Groups of Probands by Spectrally Resolved Analysis

Comparison between probands (HV, CKD without statin treatment, CKD with statin treatment) was then performed following an image separation of the two individual components (c_1_ and c_2_, respectively).

**Figure 3:**
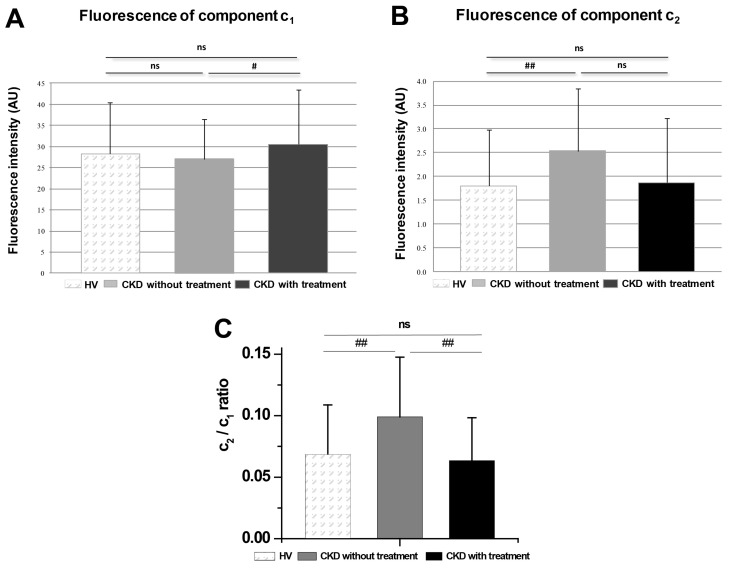
Comparison of spectrally resolved components in the group of probands. (**A**) Mean fluorescence intensity values of c_1_, (**B**) of c_2_ between groups of proband’s cells and (**C**) of the c2/c1 ratio from the HV (n = 83), CKD patients without statin treatment (n = 49) and CKD patients with statin treatment (n = 73). Data are presented as means ± SD, n = number of cells. Comparison performed with Wilcoxon test between group couples: # *p* < 0.05; ## *p* < 0.01; ns—nonsignificant difference. HV—healthy volunteers, CKD—patients with chronic kidney disease.

When a dependency of profile shapes on the group of probands was verified by estimating spectral profiles for each proband group by the same approach, no difference between the spectral components was found (data not shown). At the same time, we found that the two components responded differently to CKD vs. to its treatment with statins: while the c_1_ component showed a significant rise in response to the treatment with statins (Figure 3A), the c_2_ component, on the other hand, increased in CKD patients but remained unchanged following the treatment (Figure 3B). By comparing the proportional weight of c_1_ in the individual groups of probands, we found neither a statistically significant difference between HV and CKD without statin treatment, nor between groups of HV and CKD with statin treatment. The comparison of ratio c_2_/c_1_ showed a clear rise in the CKD group when compared to HV, but interestingly, it also showed a clear rise when compared to CKD with treatment. The gathered data point to a differential responsiveness of the individual components to a diseased state vs. to its treatment with statins.

### 3.5. Fluorescence Lifetime Imaging Microscopy (FLIM)

**Figure 4:**
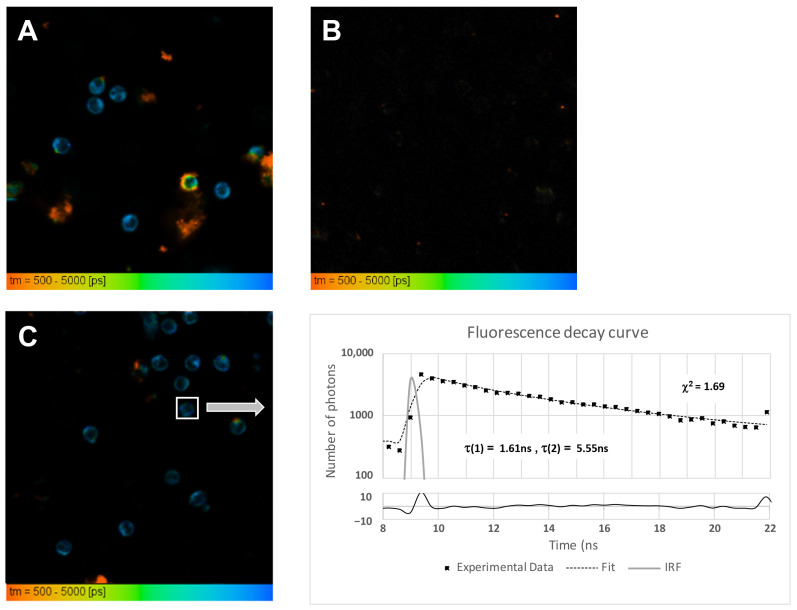
FLIM imaging of cells stained with 22-NBD-cholesterol. (**A**) Fluorescence lifetime image of cells stained with 22-NBD-cholesterol and (**B**) label-free FLIM image showing no or minimal autofluorescence in unstained PBMC. (**C**) Example of an original recording of fluorescence decay with fitting curve and goodness of fit from an individual cell. Excitation by picosecond laser with 20 MHz repetition at 473 nm, LP 505 filter, the emitted fluorescence detected by a photomultiplier HPM 100–40. Scale 500–5000 ps.

With the aim to better understand the incorporation of the 22-NBD-cholesterol to PBMC membranes, we employed FLIM. A typical FLIM image of PBMC labeled by 22-NBD-cholesterol is shown at Figure 4A. The image of unlabeled PBMC showed no or minimal endogenous fluorescence using the same experimental setting (Figure 4B), indicating that the fluorescence signals detected in these experiments can be attributed to the fluorescence of the 22-NBD-cholesterol dye only. An example of the original recording of fluorescence decay with fitting curve is shown at Figure 4C.

**Figure 5:**
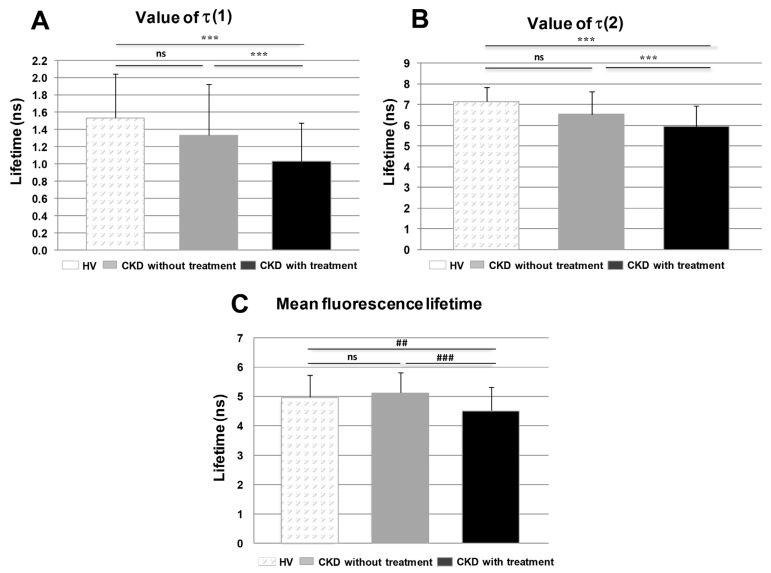
Comparison of time-resolved components in the group of probands. (**A**) Comparison of τ(1) and (**B**) of τ(2) between groups of proband cells. (**C**) The mean fluorescence lifetime calculated according to Formula 3. Data are presented as means ± SD, n = number of cells, HV (n= 28), CKD patients without statin treatment (n = 67) and CKD patients with statin treatment (n = 68). Comparison performed with *t*-test: *** *p* < 0.001. Comparison performed with Wilcoxon test between group pairs: ## *p* < 0.01; ### *p* < 0.001; ns—non-significant difference.

Recorded FLIM images underwent the fitting analysis procedure described in the Material and Methods Section: the lifetime parameters τ(1) and τ(2) (see Formula (2)) were calculated for each segmented cell and statistically evaluated for each group of samples, namely for HV, CKD with treatment and CKD without treatment (Figure 5A,B). The mean fluorescence lifetime τ_m_ (see Formula (3)) was also calculated for each group of samples (Figure 5C).

### 3.6. Comparison of Groups of Probands by Time-Resolved Analysis

When data gathered by FLIM were compared between different probands (HV, CKD without statin treatment, CKD with statin treatment), statistically significant differences were found. We uncovered that the treatment with statins significantly affected the fluorescence lifetime of the two components. More precisely, τ(1) was significantly shortened in the groups of CKD with statin treatment when compared to HV, as well as groups of cells of CKD patients without statin treatment and CKD patients with statin treatment (Figure 5A). Statistically significant changes were also observed in τ(2) when compared between groups of cells from HV and CKD patients with statin treatment and between groups of cells from CKD patients without and with statin treatment (Figure 5B). The value of amp(1) = 38 ± 9% and amp(2) = 62 ± 9% for HV changed significantly in CKD patients without statin treatment to amp(1)= 27 ± 12% and amp(2) = 73 ± 12%. In CKD patients with statin treatment, the amp(1) = 29 ± 11% and amp(2) = 71 ± 11%; these amplitudes were not significantly modified when compared to CKD without treatment, but were significantly different when compared to HV. However, observed variations in the relative contributions of the two fluorescence lifetimes depend on multiple factors that cannot be easily explained without further experimentation and need to be the subject of further studies. At the same time, statistically significant differences in the mean lifetime τ_m_ (calculated according to formula (3)) were observed for groups of HV and CKD with statin treatment, as well as from CKD patients without statin treatment and CKD patients with statin treatment patient cells (Figure 5C). The observed statistically significant differences in fluorescence lifetimes of 22-NBD-cholesterol interacting with membranes of PBMC indicate that the statin treatment affects the membranes’ quality of the PBMC from treated CKD patients.

## 4. Discussion

In this work, we employed the spectrally and time-resolved fluorescence of the 22-NBD-cholesterol to study its interaction with the cell membranes of PBMC, with the aim to evaluate changes in its optical properties in CKD patients (untreated or treated with statins) when compared to HV. We uncovered the presence of two distinct spectral forms and two lifetime components, and we found significant differences in their occurrence between the studied groups. 22-NBD-cholesterol was shown to be unable to mimic the behavior of cholesterol in lipid bilayers [27], but our work demonstrates that its interaction with PBMC allows us to employ it as a tool to distinguish patients with CKD, as well as to monitor the use of statins. The gathered results thus point to the possibility that 22-NBD-cholesterol may be employed as an additional diagnostic tool for monitoring of the presence of CKD and/or the efficiency of the treatment.

The 22-NBD-cholesterol is known for its ability to incorporate into cell membranes [28]. Based on our previous work [23] and the available literature [29,30], we developed procedures for measuring and analyzing data. The choice of the one-hour incubation time for the NBD-cholesterol with cells was made in agreement with other authors [29,30]. Spectral and time-resolved properties were studied by confocal microscopy and FLIM imaging in unstained and stained cells. We found that there is no measurable autofluorescence in the unstained PBMC that could interfere with the fluorescence of 22-NBD-cholesterol under the employed experimental conditions. The distribution of 22-NBD- cholesterol in PBMC showed localization in the plasma membrane and in the intracellular membranes of some organelles. The cell nucleus remained unstained. The same distribution of 22-NBD-cholesterol was also observed in stained human leukemia monocytic cells (THP-1), where the dye was localized in the membranes and the cell nucleus was not stained [28]. Lack of intracellular staining following treatment with digitonin, which is capable of perforating the cells in the place of the 22-NBD-cholesterol incorporation [26,31], confirmed the specific incorporation of the dye mainly into the plasma membrane.

Spectral imaging revealed the presence of two spectral forms of 22-NBD-cholesterol in PBMC. This result is in agreement with other studies [2,3,4,5,6], also describing one spectral component at 522 nm and the second one at 539 nm [2]. The first component, evenly distributed throughout the membrane bilayer, was found to be a monomeric form of the fluorophore based on its properties. The second component, based on the absorption and fluorescence properties, was attributed to the formation of dimers of the NBD probe [1,3]. Our observations in PBMC are in agreement with the studies describing the presence of two spectral forms with distinct properties.

FLIM demonstrated the presence of two fluorescence lifetime components of 22-NBD-cholesterol in PBMC. Previous studies described two lifetime components with an average τ(1) = 2.14 ns and τ(2) = 6.96 ns at 522 nm, that reached τ(1) = 2.61 ns and τ(2) = 8.52 ns at 539 nm [2]. In other studies performed on cells, the fluorescence lifetime was estimated by a one-component analysis to a mean τ = 3.8–4.2 ns [23]. The mean fluorescence lifetime in a one-component analysis of human embryonic kidney cells (HEK293) stained with 22-NBD-cholesterol was τ = 4.9 ± 0.1 ns [30]. In this study, we found the presence of two fluorophore components in our PBMC, similar to the Mukherjee and Chattopadhyay study [2]. Based on this work, it can be assumed that the spectral c_1_ (λ_max1_ = 520 nm) and the fluorescence lifetime τ(1) can represent the monomeric form 22-NBD-cholesterol, the spectral c_2_ (λ_max2_ = 552 nm) and the fluorescence lifetime τ(2) dimerized form [2]. However, the experimental results also suggest a possible association of c_1_ and the fluorescence lifetime τ(2) and vice versa of c_2_ and the fluorescence lifetime of τ(1) based on their presence in the cells. The lifetime and spectral component cannot be directly correlated; the aim of this work was not to demonstrate the connection between different spectral and time-resolved components. The reason is that such a conclusion cannot be made without an advanced physico-chemical analysis of the behavior of the 22-NBD-cholesterol dye in various environments, and it will be the subject of further studies. FLIM analysis is sensitive to environmental conditions and, consequently, the observed changes in fluorescence lifetimes may be related to structural changes, reorganization and lipid organization in plasma membrane as well as cell type [30,32].

Comparison of biochemical parameters was performed between the groups of probands (HV, CKD patients without statin treatment and CKD patients with statin treatment). The lipid profile of the studied subjects showed that CKD patients without treatment and patients treated with statins had significantly reduced serum HDL cholesterol concentrations and increased VLDL cholesterol, as well as TG concentrations compared to HV. The concentration of LDL cholesterol was increased in the CKD patients without statin treatment compared to HV. Patients treated with statins had statistically significantly reduced serum concentrations of TC and LDL cholesterol compared to patients without statin treatment. The HDL cholesterol concentration did not change significantly. These data are in accordance with the published ones [10,11,12,13,15,33]

Some of the possible indicators of changes in lipid metabolism in CKD patients are increased BMI and the elevated concentration of CRP, which are directly related to ongoing inflammation and disturbance of lipid balance [10,11,13,15,33,34]. Our CKD patients had significantly increased mean CRP values when compared to HV, although still within the reference range. The CRP concentration seemed to be higher in patients treated with statins in comparison with untreated patients; this could be due to more advanced CKD with more comorbidities in treated patients, but higher variability with such a low number of patients also cannot be excluded; moreover, the difference between both CKD patient groups was not significant.

When optical properties of the 22-NBD-cholesterol fluorescent probe were compared between the groups of probands, we observed that both spectral components (c_1_ and c_2_) uncovered by the spectrally resolved imaging were present in PBMC of all proband groups. No changes between the cells of individual subjects were observed in the spatial or spectral distribution of 22-NBD-cholesterol (data not shown). However, the fluorescence intensity of c_2_, as well as the c2/c1 ratio, were significantly increased in cells of CKD patients without statin treatment when compared to HV cells. On the other hand, the intensity of the c_1_ component was significantly altered in patients that received the statin treatment when compared to those without the treatment, while the c2/c1 returned to their original values. Such changes in the fluorescence intensity and proportion of individual components could be associated with oxidative stress in the PBMC membranes of patients with CKD [35]. We hypothesize that PBMC membranes in CKD increased the number of incorporated 22-NBD-cholesterol dimers, while the treatment affected the membrane properties in such a way that it allowed for the incorporation of a higher number of monomers. This result allows us to employ the 22-NBD-cholesterol as a potential indicator of the CKD and its treatment.

The fluorescence lifetimes of τ(1), τ(2) and the mean value of τ were determined for all three groups of PBMC probands by two-component FLIM analysis. The decrease in the mean lifetime of fluorescence τ was observed in cells of statin-treated CKD patients, along with the increase in the c_1_ and the decrease in the c_2_ representation compared with cells from untreated CKD patients. This finding may indicate the increased incorporation of 22-NBD-cholesterol in the form of monomers and the reduced number of 22-NBD-cholesterol dimers in the cell membranes of treated patients. Previously, statins were demonstrated to have the capacity to modify the lipid packaging and lipid chain order in lipid bilayers [36,37,38]. Such action can contribute to the observed effect on the fluorescence lifetimes. Additionally, increased fluorescence lifetime with increasing emission wavelength has been observed in fluorophores located in more rigid regions of the membrane with limited mobility and higher cholesterol content [2,32]. It is known that the amount of cholesterol in cell membranes directly modulates their rigidity and that the increased formation of cholesterol dimers is associated with a higher cholesterol content in membranes [3]. An increased amount of cholesterol dimers in the membrane also prolongs fluorescence lifetime in the cell [2]. The observed shortening of the fluorescence lifetimes of the 22-NBD-cholesterol in CKD patients following statin treatment is in agreement with the reduced rigidity of the membrane and lower cholesterol content. Although an exact mechanism needs to be determined by future studies, FLIM allowed us to demonstrate that the 22-NBD-cholesterol fluorescence is affected in cells from statin-treated CKD patients and can thus be used for monitoring of the treatment efficiency.

## 5. Conclusions

In conclusion, the aim of this contribution was to monitor the interactions of a fluorescent probe, the 22-NBD-cholesterol, with PBMC membranes in HV and CKD patients. The fluorescence of 22-NBD-cholesterol was detected on the cell membranes or intracellularly, excluding the nuclear region. The analysis of spectral imaging results uncovered the presence of two distinct spectral forms of 22-NBD-cholesterol with the differential variation of their occurrence between HV and CKD patients treated with statins in comparison to patients without statin treatment. FLIM revealed the presence of two lifetime components with their significant variations in CKD patients treated with statins. Described changes in optical properties of the 22-NBD-cholesterol fluorescent dye in PBMC of both the untreated and the statin-treated CKD patients indicate the capacity of the probe to monitor the disturbance of lipid metabolism in CKD, as well as the efficiency of the treatment with statins.

## Figures and Tables

**Table 1:** Clinical and biochemical parameters.

	HV	CKD without Statin Treatment	CKD with Statin Treatment
**BMI** [kg/m^2^]	22.5 ± 3.4	28.7 ± 6.5 *	28.8 ± 5.1 *
**eGFR** [mL/s]	1.6 ± 0.2	0.71 ± 0.23 ***	0.58 ± 0.33 ***
**TC** [mmol/L]	4.3 ± 0.59	0.43 ± 1.47	3.73 ± 0.88 #
**HDL** [mmol/L]	1.55 ± 0.26	0.92 ± 0.22 **	0.86 ± 0.05 ***
**LDL** [mmol/L]	2.16 ± 0.6	3.39 ± 0.92	0.58 ± 0.78 ##
**VLDL** [mmol/L]	0.52 ± 0.23	1.11 ± 0.43	1.17 ± 0.23 ***
**TG** [mmol/L]	1.3 ± 0.32	2.44 ± 0.94 *	2.94 ± 1.08 **
**CRP** [mg/L]	0.5 ± 0.2	4 ± 4 **^+^**	8 ± 7 **^+^**

HV: healthy volunteers, CKD: patients with chronic kidney disease, TC: total cholesterol, HDL: high-density lipoprotein cholesterol, LDL: low-density lipoprotein cholesterol, VLDL: very low-density lipoprotein cholesterol, TG: triglycerides, CRP: C-reactive protein. Values are means ± standard deviation. Statistical evaluation with Student’s *t*-test: HV to CKD patients without statin treatment or HV to CKD patients with statin treatment: * *p* < 0.05; ** *p* < 0.01; *** *p* < 0.001. Statistical evaluation with Wilcoxon test: CKD patients without statin treatment to CKD patients with statin treatment: # *p* < 0.05; ## *p* < 0.01. Statistical evaluation with Wilcoxon test: HV to CKD patients without statin treatment or HV to CKD patients with statin treatment: **^+^** *p* < 0.05.

## Data Availability

Data are available on request in an Omero repository at http://microscopy.mlc.sk/omero (accessed on 12 April 2019).

## Sample Availability

Samples are not available from the corresponding authors.
